# Fabrication of a Selective Sensor Amplification Probe Modified with Multi-Component Zn_2_SnO_4_/SnO_2_ Heterostructured Microparticles as a Robust Electrocatalyst for Electrochemical Detection of Antibacterial Drug Secnidazole

**DOI:** 10.3390/ma14216700

**Published:** 2021-11-07

**Authors:** Ramaraj Sukanya, Karuppaiah Balamurugan, Shen-Ming Chen, Ramachandran Rajakumaran, K. Muthupandi, Jae-Jin Shim, Carmel B. Breslin

**Affiliations:** 1 Electroanalysis and Bioelectrochemistry Lab, Department of Chemical Engineering and Biotechnology, National Taipei University of Technology, No. 1, Section 3, Chung-Hsiao East Road, Taipei 106, Taiwan; sukanyaram33@gmail.com (R.S.); balamurugnmdu7@gmail.com (K.B.); rajvsragzz@gmail.com (R.R.); 2 Department of Chemistry, Mannar Thirumalai Naicker College, Madurai 625004, India; k.muthupandi26@gmail.com; 3 School of Chemical Engineering, Yeungnam University, Gyeongsan 38541, Gyeongbuk, Korea; jjshim@yu.ac.kr; 4 Department of Chemistry, Maynooth University, W23F2H6 Maynooth, Co. Kildare, Ireland

**Keywords:** ternary metal oxides, heterostructure, electrochemical sensor, secnidazole, Zn_2_SnO_4_/SnO_2_ MPs

## Abstract

In this study, we synthesized heterostructured zinc stannate/tin oxide microparticles (ZTO/TO MPs) by a simple coprecipitation method and used them as an effective electrode material for the electrochemical detection of the antibacterial drug secnidazole (SCZ). The as-prepared ZTO/TO MPs were characterized by XRD, Raman, FE-SEM, HR-TEM, EDX, and XPS analyses. The physiochemical studies clearly proved that the fabricated ZTO/TO MPs were formed in a heterostructure phase without other impurities. A glassy carbon electrode modified with the synthesized ZTO/TO MPs showed an excellent and improved electrocatalytic activity in the electrochemical reduction of SCZ. Using differential pulse voltammetry (DPV), an impressive linear calibration range, extending from 0.01 to 193 μM, was observed, coupled with a detection limit of 0.0054 μM and a sensitivity of 0.055 μA/μM. In addition, the ZTO/TO MPs/GCE showed very good selectivity for the detection of SCZ in the presence of a number of biological, inorganic, and structurally related compounds. Finally, the ZTO/TO MPs/GCE was investigated for the analysis of SCZ in human blood serum samples. A very good recovery was obtained when spiking the blood serum with SCZ, highlighting the good applicability of the ZTO/TO MPs/GCE for the electrochemical analysis of SCZ in complex biological samples.

## 1. Introduction

Multifunctional metal oxides with multivalent ions form an extensive collection of various nanostructured functional materials with numerous applications in science and technology [1]. In this series, binary metal oxides have been intensively studied, and research on specially designed ternary oxide semiconductors has recently received increasing attention. In particular, semiconducting ternary metal oxides are currently of great interest due to their potential technological applications in photocatalysis [2], solar cells [3], and lithium-ion batteries [4] as they exhibit a tunable band gap (3.1–3.9 eV), high electron transport, and high chemical stability. Specifically, the combination of zinc oxide (ZnO) and tin oxide (SnO_2_), to give zinc stannate/tin oxide (ZTO/TO), is characterized by a good conductivity, low toxicity, and high thermal and chemical stability, and it makes a significant contribution as an electrode material in several applications [2,3]. The main crystal structure of ZTO is classified into two combinations: zinc stannate (ZnSnO_3_) with a perovskite structure and dizinc stannate (Zn_2_SnO_4_) with an inverse spinel structure [5]. Zinc stannate is a ternary n-type semiconducting oxide that exhibits multifunctional properties, including a wide band gap (3.6 eV), tunable working potential, high electrical conductivity (10^−2^–10^−3^ Ω cm), high electron mobility (20–50 cm^2^/Vs), high chemical stability, slow recombination rate between electrons and holes, and low adsorption capacity, making it a suitable material for various electrochemical energy storage, conversion, and sensing applications [6,7,8,9]. The properties of ZTO/TO mainly result from the cubic/tetragonal combination of ZTO/TO, which consists of both tetrahedral and octahedral sites, where the tetrahedral sites are occupied by Zn^2+^ cations, while the octahedral sites are occupied by randomly arranged Zn^2+^ and Sn^4+^ cations in equal percentage ratios [10]. Moreover, this ZTO/TO combination contains oxygen vacancies (*V*_O_) (lattice-oxygen vacancies-interstitials or oxygen interactions), which have a significant effect on the electronic and electrochemical properties, giving rise to enhanced electron transfer reactions [11,12]. This aspect has been demonstrated by Li et al. by comparing the electrochemical behaviour of hybrid ZTO/TO for various applications, such as photocatalysis, solar cells, and sensing [13], while it has also been shown by Wang et al. that the ZTO/TO hybrid facilitates the electrochemical reduction of carbon dioxide [14]. Moreover, ZTO/TO exhibits properties such as a large active surface area, high diffusion path, and high Lewis acid−base character, which also contributes to an improved electrochemical sensing performance by transporting electrons or ions through the electrolyte solution via the diffusion path of the cubic lattice [15]. Considering all these characteristics, we believe that the ZTO/TO hybrid is a good candidate for the electrochemical detection of nitro-based drug compounds.

Secnidazole (1-(2-methyl-5-nitro-1H-imidazol-1-yl)propan-2-ol (SCZ)) is a well-known antibacterial drug and is structurally related to the family of commonly used 5-nitroimidazoles, such as metronidazole and tinidazole [16]. These drugs share a spectrum of activity against anaerobic microorganisms and are particularly effective in the treatment of amoebiasis, giardiasis, trichomoniasis, and bacterial vaginosis. Due to its antimicrobial activity, it also inhibits the growth of certain anaerobic protozoa such as *Trichomonas, Vaginalis,* and *Entamoeba histolytica* [17]. In addition, SCZ has been shown to be effective against *Dientamoeba fragilis*, which can cause diarrhoea, and has been prescribed for the treatment of periodontitis. The mechanism of action of these nitroimidazoles is mainly based on the electron accepting ability of the nitro group [17]. This compound is metabolized in vivo to the corresponding radical anion. The nitro radical anion then induces DNA damage in various cell systems, including anaerobic and microaerophilic bacteria and protozoa. This selective effect depends on the standard potential of the R-NO_2_/R-NO_2_^∙^^−^ system of nitro and anion radicals [17]. The activity and pharmacokinetics of these agents have been explained in numerous reviews. In addition, the oral administration of SCZ results in a longer terminal elimination half-life than other commonly used nitro compounds [18]. Therefore, pharmaceutical quality control and therapeutic monitoring of SCZ are crucial for confirming toxicity levels, drug−drug interactions, and guiding the discontinuation of other antiparasitic drugs. Therefore, it is necessary to develop a more effective and sensitive method to determine the SCZ concentration in drugs or biological samples. To date, various analytical methods have been used to determine SCZ in biological samples, including visible and ultraviolet spectrophotometry [19], high-performance liquid chromatography (HPLC) [20], gas chromatography [21], and liquid chromatography−tandem mass spectrometry [22]. However, many of these analytical techniques are time-consuming and require expensive equipment, constant maintenance, and a high level of user expertise. Electrochemical methods, such as cyclic voltammetry and differential pulse voltammetry, have received considerable attention for measuring target analytes, such as drug molecules, due to their fast response time and high sensitivity [18]. Moreover, they only require compact equipment. For this reason, we believe that an electrode modified with ZTO/TO MPs is a good choice for the electrochemical sensing of SCZ. 

There are very few sensors that have been reported for the electrochemical detection of SCZ. Glassy carbon and mercury substrates have previously been employed. For example, Radi et al. [17] employed DC and AC polarography with a dropping mercury electrode to analyse the SCZ content in tablets, while El-Sayed et al. [23] used cathodic adsorptive stripping voltammetry for the analysis of SCZ with dropping mercury and glassy carbon electrodes. In this study, we synthesized ZTO/TO MPs using a simple and inexpensive coprecipitation method. Then, various characterization techniques such as XRD, Raman, FE-SEM, HR-TEM, and XPS analyses were performed to better understand the crystalline nature, surface chemistry, and bonding interactions. Subsequently, a GCE was modified with the ZTO/TO MPs and was employed in the electrochemical analysis and detection of SCZ with CV and DPV techniques. The electrochemical results showed that the determination of SCZ with ZTO/TO MPs/GCE has a low detection limit, high selectivity and sensitivity, and this modified electrode is also very suitable for the electrochemical detection of SCZ in practical applications.

## 2. Materials and Methods

Zinc nitrate hexahydrate (Zn(NO_3_)_2_⋅6H_2_O), sodium stannate trihydrate (Na_2_SnO_3_·3H_2_O), ethylene glycol, urea (CO(NH_2_)_2_), secnidazole, sodium phosphate dibasic anhydrous (Na_2_HPO_4_), sodium phosphate monobasic dihydrate (NaH_2_PO_4_⋅2H_2_O), and the other required chemicals and reagents were obtained from Sigma-Aldrich (Taipei, Taiwan). All of the reagents and chemicals used in this work were of analytical grade, and deionized (DI) water was used to prepare the required solutions. A phosphate buffer solution (0.05 M (PBS)) was employed as the supporting electrolyte, which was prepared by dissolving sodium phosphate dibasic anhydrous and sodium phosphate monobasic dihydrate in DI water. Aqueous sodium hydroxide and hydrochloric acid solutions were used to adjust the pH of the buffer solutions.

**Scheme 1:**
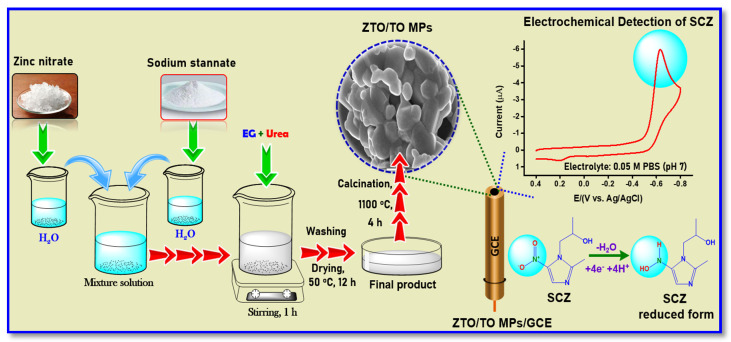
The overall synthesis procedure of the ZTO/TO MPs sensor and its electrochemical application in the detection of SCZ.

The co-precipitation method was used for the synthesis of the ZTO/TO MPs. In a typical synthesis, 30 mL solutions of 200 mM Na_2_SnO_3_.3H_2_O and 200 mM of Zn(NO_3_)_2_.6H_2_O were prepared individually and mixed with 50 mL DI water in a beaker (200 mL). With stirring, 7 mL of ethylene glycol and 5 g (in 10 mL of H_2_O) of CO(NH_2_)_2_ were then added to the beaker and stirred for 1 h (800 rpm) at room temperature. After 1 h, a white coloured precipitate was obtained, which was washed three times with water and then with ethanol to remove the impurities present in the product. Then, the final precipitate was placed in an air oven and stored at 50 °C for 12 h. Finally, the ZTO/TO MPs were obtained after the crystallization process in a muffle furnace at 1100 °C for 4 h. The overall synthesis procedure of the ZTO/TO MPs and a schematic of their electrochemical sensing properties are shown in Scheme 1.

Prior to modification, the surface of GCE (working area = 0.07 cm^2^) was polished with alumina slurry (0.05 µm) and washed with DI water. The ZTO/TO MPs suspension (5 mg/mL) was prepared using ultra-sonication for 20 min to ensure uniform distribution of the MPs. Then, 6 µL of the ZTO/TO MPs suspension was drop casted onto the cleaned GCE surface and dried in an air oven for 10 min. Finally, the ZTO/TO MPs/GCE was used for the electrochemical experiments.

To investigate the crystallographic phase and purity of the as-prepared ZTO/TO MPs, powder X-ray diffraction (PXRD) was performed in the range of 2θ = 10 to 90° using a DMAX-IIIA diffractometer (λ = 0.154 nm) (Rigaku, Tokyo, Japan). Raman spectroscopy (DONGWO, Gwangju-Si, Korea, Ramboss 500i micro-Raman/PL spectroscopy) was used to analyse the molecular interactions. Morphological characterization, composition, and elemental distribution were analysed by field emission scanning electron microscopy (ZEISS Sigma 3000; FE-SEM, Oberkochen, German), high-resolution transmission electron microscopy (JEOL 2100F; HR-TEM, Tokyo, Japan), and energy-dispersive X-ray spectroscopy (EDX). The presence of elements and the binding energies were analysed by X-ray photoelectron spectroscopy ((XPS) Thermo-scientific multi-lab 2000, Waltham, MA, USA).

Electrochemical analyses were performed with CHI potentiostats. (Austin, TX, USA) Cyclic voltammetry (CV) experiments were carried out with a CHI1205C, while differential pulse voltammetry (DPV) was performed with a CHI900 potentiostat. The optimized DPV parameters were as follows: potential window from +0.4 to −0.8 V, amplitude of 0.05 V, pulse width of 0.05 s, and a pulse period of 0.2 s. All of the electrochemical experiments were performed in a 20 mL electrolytic cell containing 10 mL of N_2_-saturated PBS solution with a standard three-electrode system, with the ZTO/TO MPs/GCE as the working electrode, KCl-saturated Ag/AgCl as the reference electrode, and a platinum wire as the counter electrode.

## 3. Result and Discussion

### 3.1. X-ray Diffraction and Raman Studies 

**Figure 1:**
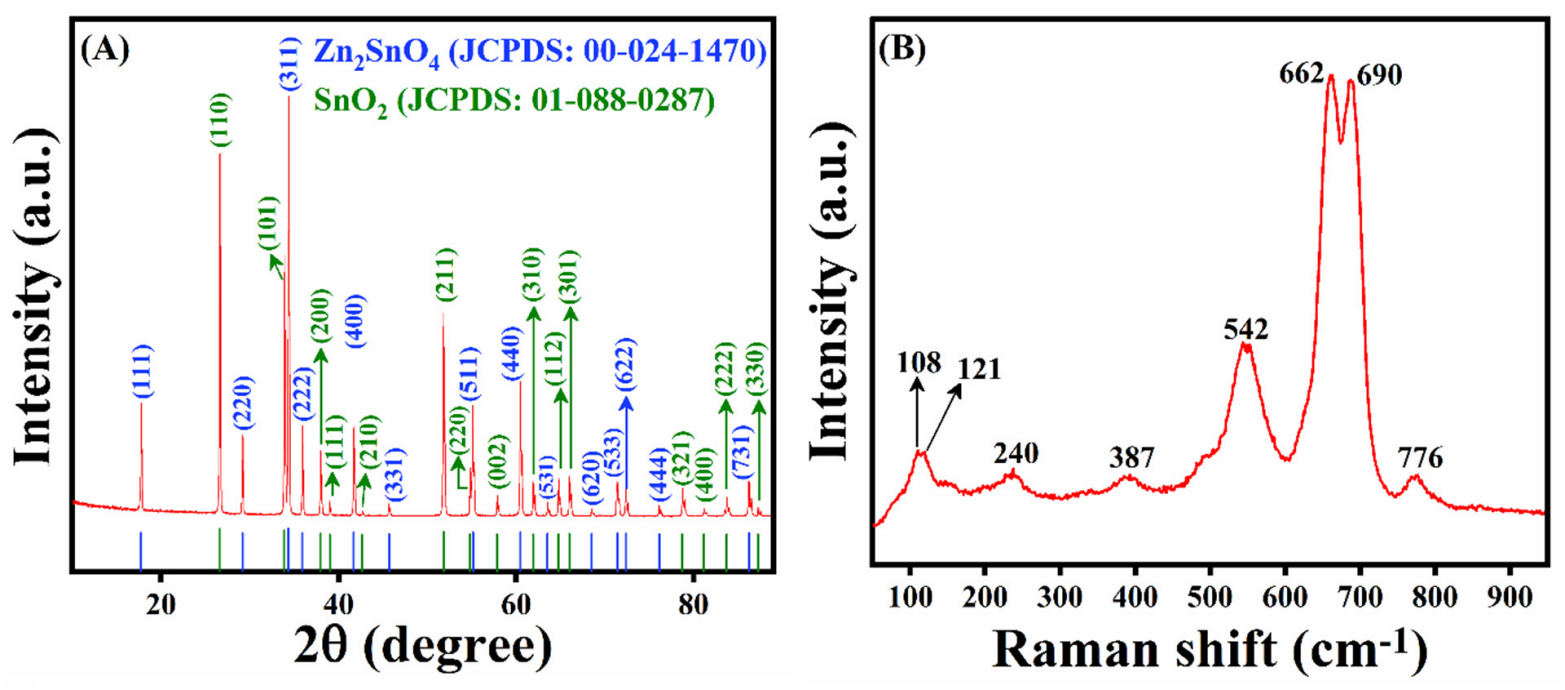
(**A**) XRD pattern and (**B**) Raman spectra of ZTO/TO MPs.

The phase purity of the as-prepared ZTO/TO MPs was investigated by powder XRD analysis. Figure 1A shows the XRD pattern of the ZTO/TO MPs. Several diffraction peaks at an angle of 17.7°, 29.1°, 34.2°, 35.9°, 41.6°, 45.6°, 55.1°, 60.4°, 63.5°, 68.4°, 71.3°, 72.3°, 76.1°, and 86.2° were found, corresponding to the (111), (220), (311), (222), (400), (331), (511), (440), (531), (620), (533), (622), (444), and (731) planes, respectively. These peaks are in good agreement with the inverse spinel structure of Zn_2_SnO_4_. The diffraction peaks at an angle of 26.5°, 33.8°, 37.9°, 38.9°, 42.6°, 51.7°, 54.7°, 57.8°, 61.8°, 64.7°, 65.9°, 78.8°, 81.1°, 83.7°, and 87.2° are consistent with the (110), (101), (200), (111), (210), (211), (220), (002), (310), (112), (301), (321), (400), (222), and (330), planes of the tetragonal rutile phase of SnO_2_. Furthermore, the peak positions and their relative intensities can be compared with the standard data of Zn_2_SnO_4_ and SnO_2_, which show that the crystal structure consists of a combination of face-centred cubic structures with space group fd-3m (JCPDS: 00-024-1470) and tetragonal rutile (JCPDS: 01-088-0287) [24]. It can be seen that all the peak positions are characteristic of the pure ZTO/TO compound. There are no other impurity peaks, such as ZnO and ZnSn(OH)_6_, in the XRD patterns, except those assigned to binary oxide phases. This XRD analysis clearly shows that the heterostructured ZTO/TO MPs were successfully formed by the simple coprecipitation method.

Additional chemical information was obtained using Raman analysis and a representative spectrum is shown in Figure 1B. Based on group theory, the Raman spectra of pure Zn_2_SnO_4_ and SnO_2_ can be categorized into seven modes, which are theoretically assigned to T_2u_, B_1g_, F_2g_, E_g_, A_2u_, B_2g_, and A_1g_ symmetries. Considering the Raman spectrum, the peak at 108 cm^−^^1^ can be assigned as the silent T_2u_ mode, while the peak at 121 cm^−^^1^ corresponds to the B_1g_ mode. The wavenumber at 240 cm^−^^1^ is attributed to the E(LO) phonon mode and the peak at 387 cm^−^^1^ is associated with the F_2g_ mode. In addition, the band at 542 cm^−^^1^ is consistent with the internal vibrations of the oxygen tetrahedron. It is worth highlighting that the mode with the highest intensity at 662 cm^−^^1^ can be attributed to the symmetric stretching of Zn-O bonds in the SnO_4_ tetrahedra of the inverse Zn_2_SnO_4_. The weak Raman band at 690 cm^−^^1^ can be assigned to the A_2u_ mode, while the peak at 776 cm^−^^1^ can be attributed to the first order E_g_ (translational), A_1g_ (symmetric Sn-O stretching), and B_2g_ (asymmetric Sn-O stretching) active Raman vibrational modes of SnO_2_ [25,26,27]. All these Raman modes confirm that the ZTO/TO MPs exist exclusively in a heterostructure. 

### 3.2. Morphological Investigation Using FE-SEM and HR-TEM Analysis

**Figure 2:**
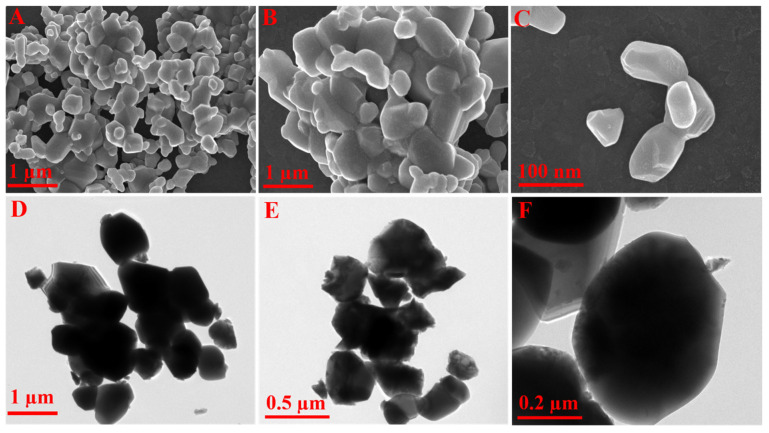
(**A**–**C**) FE-SEM images of ZTO/TO MPs and (**D**–**F**) HR-TEM images of ZTO/TO MPs.

**Figure 3:**
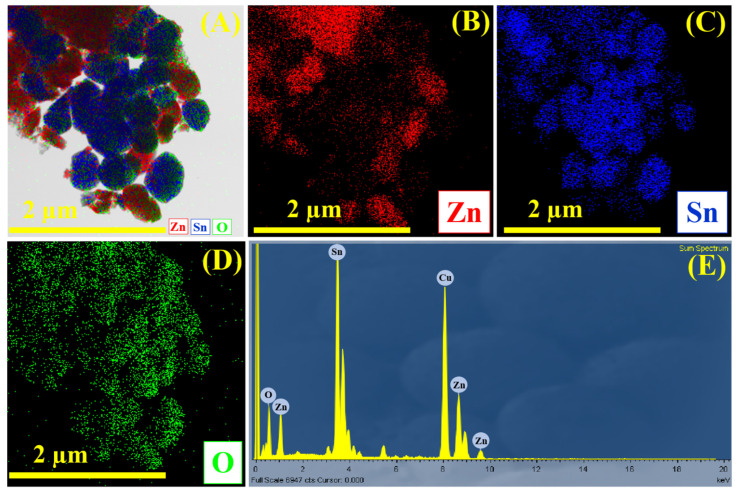
FE-SEM elemental mapping of Zn, Sn, and O (**A**); elemental mapping of (**B**) Zn, (**C**) Sn, and (**D**) O; and (**E**) EDX spectrum of ZTO/TO MPs (Cu is from the copper grid).

In addition, FE-SEM and HR-TEM studies were performed to study the morphology of the MPs, and these micrographs are shown in Figure 2A–F, indicating that the ZTO/TO MPs have the shape of a stone-like structure. The high magnification FE-SEM image in Figure 2C shows that the diameters of both the ZTO and TO MPs, with a rough surface, are in the range of several micrometres. The HR-TEM images shown in Figure 2D–F indicate that the synthesized products have heterostructures with diameters of ~0.2 μm. Furthermore, EDX and elemental analyses were carried out to confirm the presence of Zn, Sn, and O. As shown in Figure 3A–E, the EDX spectrum of the ZTO/TO MPs indicates the presence of the Zn, Sn, and O elements. The elemental mapping images presented in Figure 3A–D show that the Zn (red), Sn (blue), and O (green) elements are uniformly distributed throughout the ZTO/TO. These data show that the MPs have a dense and non-uniform structure, which may facilitate the diffusion of electrolytes, while the proximity of the adjacent SnO_2_ particles may provide a transport channel for the transfer of electrons, leading to a reduction in the electron transfer resistance.

### 3.3. X-ray Photoelectron Spectroscopy Analysis

**Figure 4:**
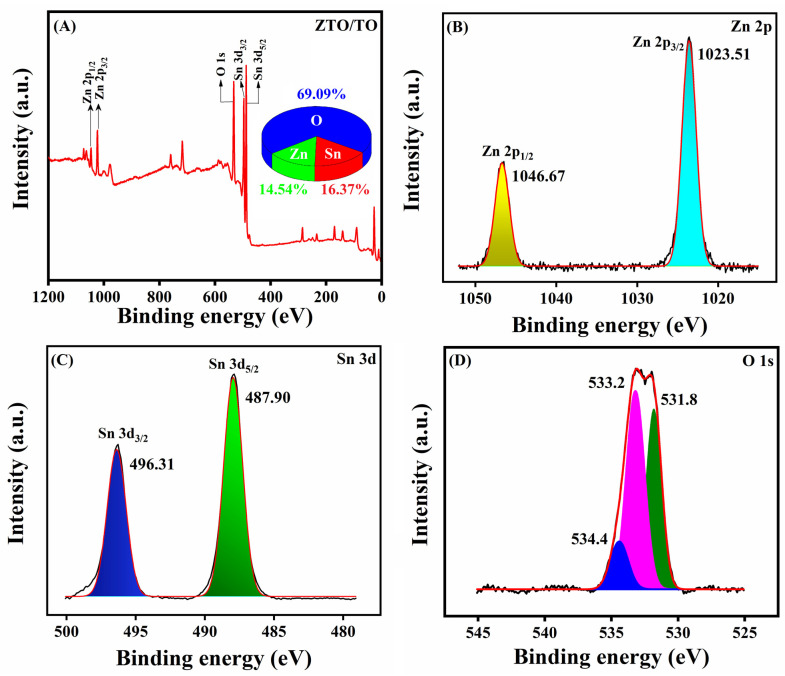
(**A**) XPS survey spectra of ZTO/TO MPs and high-resolution XPS spectra of (**B**) Zn 2p (**C**) Sn 3d, and (**D**) O 1s.

X-ray photoelectron spectroscopy (XPS) was performed to confirm the chemical composition and electronic state of the as-prepared ZTO/TO heterostructures. Figure 4A shows the overall spectrum of the ZTO/TO MPs indicating the presence of Zn, Sn, and O. In addition, the inset (Figure 4A) shows the elemental composition of the ZTO/TO MPs and this composition agrees well with the obtained XRD phase formula of Zn_2_SnO_4_/SnO_2_. The high-resolution XPS spectra of the Zn 2p, Sn 3d, and O 1s elements are shown in Figure 4B–D. The core spectrum of Zn shows peaks at 1046.6 and 1023.5 eV, corresponding to the 2p_1/2_ and 2p_3/2_ Zn^2+^ states [28,29,30]. Figure 4C shows the core spectrum of Sn, and the two strong peaks at 487.9 and 496.3 eV can be attributed to the binding energies of Sn 3d_5/2_ and Sn 3d_3/2_, respectively, which are characteristic of the Sn 3d state. Figure 4D shows the core level spectrum of the O 1s peak, which can be split into three peaks with binding energies of 531.8, 533.2, and 534.4.7 eV. The peaks at 531.8 eV and 533.2 eV can be ascribed to the lattice O in metal oxygen bonds (Zn-O, and Sn-O). The peak at 534.4 eV can be assigned to surface oxygen species such as OH or O coordination in metal oxides [31]. Again, this XPS analysis clearly confirms the presence of the heterostructured ZTO/TO MPs. 

### 3.4. Electrochemical Reduction of SCZ

**Scheme 2:**
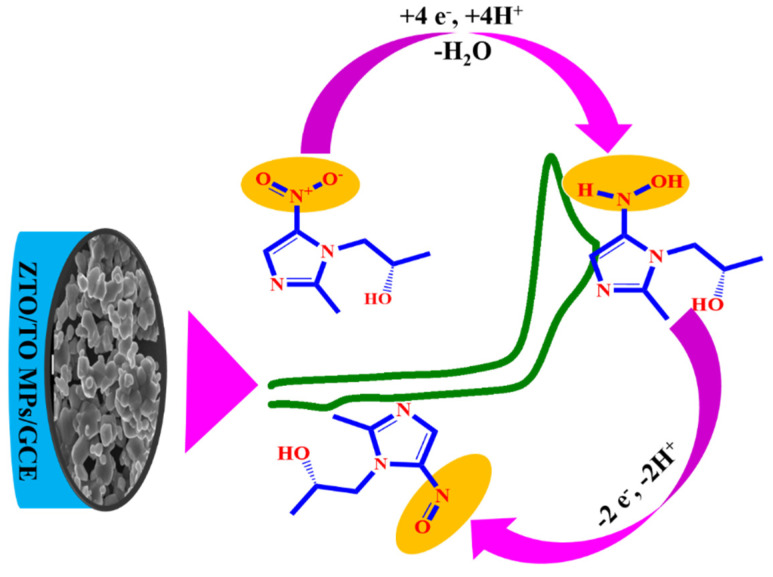
The electrochemistry of SCZ at ZTO/TO MPs/GCE, showing the reduction mechanism of SCZ and the conversion of the electrogenerated hydroxylamine group (SCZ-NHOH) to the nitroso group (SCZ-NO).

**Figure 5:**
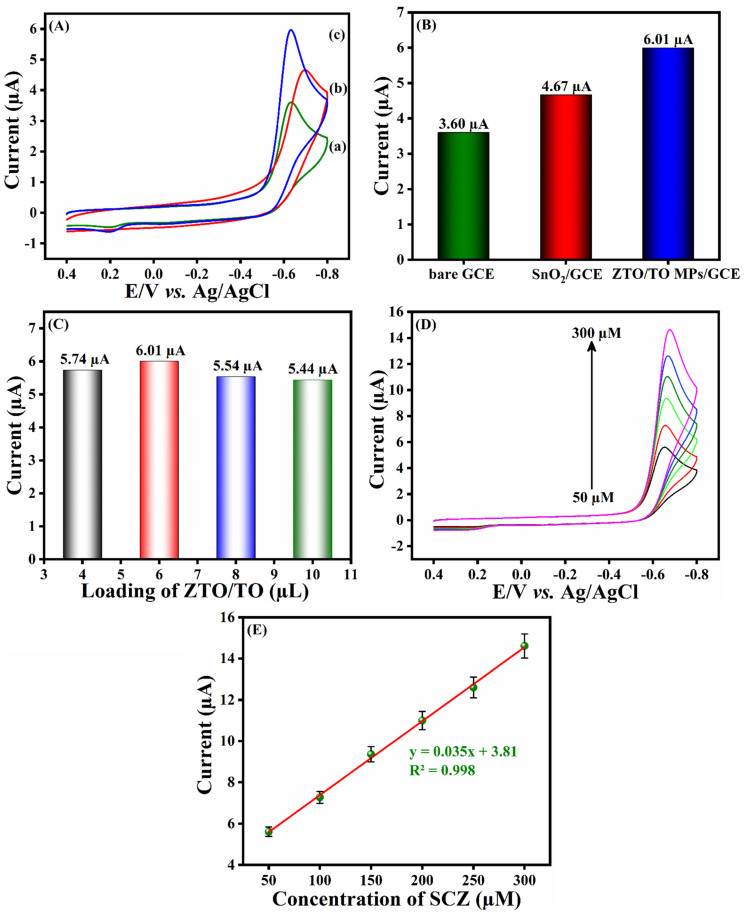
(**A**) CVs at (a) bare GCE, (b) SnO_2_/GCE, and (c) ZTO/TO MPs/GCE for 50 µM SCZ. (**B**) Comparative bar charts for the cathodic current of SCZ at bare GCE, SnO_2_/GCE, and ZTO/TO MPs/GCE. (**C**) The reduction peak current of 50 µM SCZ at ZTO/TO MPs with different catalyst loadings. (**D**) CVs of ZTO/TO MPs/GCE with different concentrations of SCZ (50–300 µM). (**E**) Cathodic current as a function of the SCZ concentration. All of the electrochemical experiments were recorded at 50 mV/s in deoxygenated 0.05 M PBS in the potential region between +0.4 to –0.8 V.

The electrocatalytic activity of ZTO/TO MPs/GCE towards the reduction of SCZ was analysed using the CV technique at a sweep rate of 50 mV/s by cycling between the potential limits of +0.4 to −0.8 V. Figure 5A shows the CV responses of 50 µM SCZ at the bare GCE, SnO_2_/GCE, and ZTO/TO MPs/GCE. The unmodified GCE, SnO_2_/GCE, and ZTO/TO MPs/GCE electrodes show a clear reduction wave in the forward sweep and a small oxidation peak in the reverse sweep. The peak obtained in the forward sweep is related to the electrochemical reduction of SCZ-NO_2_ to SCZ-NHOH with the transfer of 4e^−^ and 4H^+^. The small peak in the reverse scan represents the conversion of the hydroxylamine group (SCZ-NHOH) to the nitroso group (SCZ-NO), as illustrated in Scheme 2 [32,33]. As shown in Figure 5A, the SCZ is effectively reduced at the bare GCE, giving rise to a cathodic peak current of 3.60 µA with the reduction peak potential centred at −0.633 V. Interestingly, when the GCE is modified with SnO_2_, it shows a higher peak current than the bare GCE, but the reduction peak potential is shifted to more negative potentials. However, a higher peak current of 6.01 µA is obtained for the ZTO/TO MPs/GCE. Compared with bare GCE, when the ZTO/TO MPs and SnO_2_/GCE were used as modifiers for GCE, the peak current for the reduction of SCZ was increased by factors of approximately 2.3 and 1.2, respectively. This is more clearly illustrated in the comparison diagram shown in Figure 5B. These results clearly show that the GCE modified with ZTO/TO MPs acts as a good electron conducting mediator between the electrode surface interface and the electrolyte, and facilitates the electrochemical reduction of SCZ. Figure 5C shows the bar graph of the different loadings of ZTO/TO MPs on GCE. From this, it can be seen that a higher peak current is obtained when 6 µL of the dispersed ZTO/TO MPs is employed. Therefore, 6 µL of ZTO/TO MPs was chosen as the optimal amount of catalyst.

The influence of the SCZ concentration is shown in Figure 5C, where the CV curves are presented for ZTO/TO MPs/GCE cycled in different concentrations of SCZ, ranging from 50 to 300 µM, at a sweep rate of 50 mV/s. It is clear from this figure that the peak currents increase with increasing the SCZ concentration, and, as shown in Figure 5D, a linear relationship exists between the concentration and peak current. The corresponding linear regression equation was obtained as y = 0.035x + 3.81, with a correlation coefficient of R^2^ = 0.998, indicating good linearity. Again, these data show that the ZTO/TO MPs facilitate the electrochemical reduction of the SCZ molecules, giving a linear calibration curve with a well resolved peak at 50 µM SCZ. Thus, the ZTO/TO MPs/GCE appears to be a suitable electrode material for the electrochemical detection of SCZ. 

### 3.5. Optimization of the pH and Scan Rate Studies

**Figure 6:**
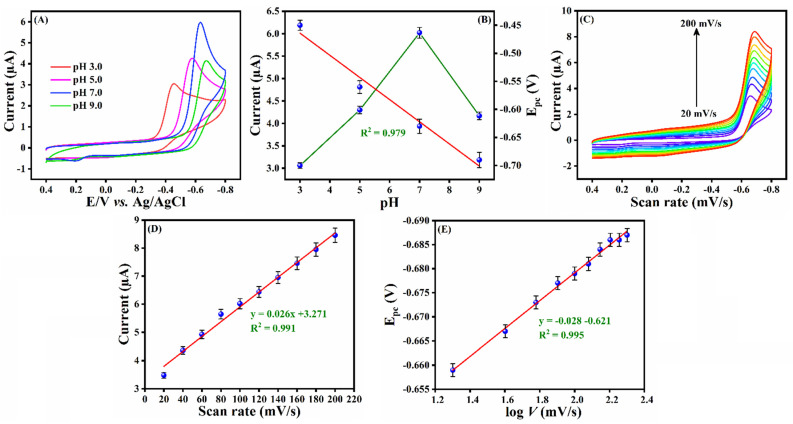
Data recorded in 50 μM SCZ in 0.05 M PBS at ZTO/TO MPs/GCE (**A**) at pH values of 3.0, 5.0, 7.0, and 9.0 at 50 mV/s; (**B**) peak current plotted as a function of pH (green) and the peak potential as a function of pH (red); (**C**) CVs at different sweep rates of 20–200 mV/s; (**D**) peak current plotted as a function of scan rate; and (**E**) peak potential as a function of the logarithm of scan rate (*E*_pc_ vs. log *V*).

The presence of protons can affect the electrochemical properties of electroactive materials and they can also participate in the electron transfer step during the oxidation or reduction of organic molecules. The impact of the electrolyte pH on the reduction of SCZ at ZTO/TO MPs/GCE was studied using CV. Representative voltammograms, recorded in 50 µM SCZ in different PBS solutions of varying pH values of 3.0, 5.0, 7.0, and 9.0 are presented in Figure 6A. It can be seen that the cathodic peak current increases with an increase of pH from 3.0 to 7.0, and then decreases at pH 9.0, due to the lack of protons, which are required during the reduction of SCZ (Scheme 2). The pKa of SCZ has been reported as 1.13 [34], which is well below a pH of 3.0, indicating that the SCZ molecule is neutral between pH values of 3.0 and 9.0. The somewhat lower peak currents observed at a pH of 3.0 may be connected to the stability of the zinc and tin oxide phases in this acidic environment. According to Pourbaix diagrams, the thermodynamically stable phases in acidic solutions are normally the solvated cations, while the oxide phases exhibit very good stability at somewhat higher pH values in the vicinity of 5.0 to 8.0. The highest peak current was observed at a pH of 7.0 (Figure 6B, green trace), and this optimized neutral electrolyte was used in all further electrochemical experiments. 

In addition, the peak potential of the reduction wave of SCZ shifted to more negative values when the pH was varied from 3.0 and 9.0, implying that protons play a role in the reduction of SCZ. A linear correlation plot between the pH of the electrolyte and the peak potential was generated and this plot is presented in Figure 6B (red line). The corresponding linear regression equation is provided in Equation (1).

*E*_pc_ = 0.041 pH + 0.334; R^2^ = 0.979 (1)

The computed slope agrees approximately with the theoretical slope of 0.059 m/n, in which m represents the number of protons transferred and n corresponds to the number of electrons transferred. An ideal Nernstian response will only exist when thermodynamic equilibrium is achieved (reversible systems with fast kinetics) and the deviation observed is probably connected with the irreversible redox behaviour of SCZ. However, this is consistent with the transfer of equal numbers of protons and electrons during the electrochemical reduction of SCZ at ZTO/TO MPs/GCE, as illustrated in Scheme 2. 

Further information on the reduction of SCZ at the ZTO/TO MPs/GCE was obtained using a scan rate study. In this case, the sweep rate was varied from 20 to 200 mV/s. Typical data recorded at different sweep rates for the GCE modified with the ZTO/TO MPs in a 0.05 M PBS solution containing 50 µM SCZ are presented in Figure 6C. It can be clearly seen that as the sweep rate increases, the reduction peak current of SCZ also increases. Moreover, a linear relationship between the peak current and scan rate was obtained, and the corresponding linear plot is shown in Figure 6D along with the linear regression equation *I*_pc_ (µA) = 0.026 *V* (mV/s) + 3.271, and a correlation coefficient of R^2^ = 0.991, where *V* represents the scan rate. This linear relationship between the peak current and scan rate indicates that the electrochemical reduction of SCZ at ZTO/TO MPs/GCE is an adsorption-controlled process. 

As evident from Figure 6C, the peak potential associated with the reduction of SCZ is shifted to more negative values when increasing the sweep rate from 20 to 200 mV/s. On plotting the peak potential as a function of the logarithm of the scan rate, a linear plot was obtained, as shown in Figure 6E. The linear regression equation was found to be *E*_pc_ (µA) = −0.028*V* (mV/s) − 0.621, with a correlation coefficient of R^2^ = 0.995. According to the Laviron equation [35], the slope of the linear plot between *E*_pc_ and log (*V*) can be expressed in Equation (2), where *R* and *F* are the universal constants (*R* = 8.314 J/K mol and *F* = 96,485.33 s A/mol) and *T* represents the thermodynamic temperature.

*E*_pc_/log(*V*) = −2.3 *RT*/*αnF* (2)

Setting *T* at 298.15 K, the *αn* value was computed as 2.2, and applying the *α* value of 0.55 (for irreversible electrochemical reactions), the number of electrons involved in the electrochemical reduction of SCZ was estimated as 4.0. This is in very good agreement with the electrochemical conversion of SCZ-NO_2_ to SCZ-NHOH at the ZTO/TO MPs/GCE, where the reduction is accompanied by the transfer of 4e^−^ and 4H^+^, as shown in Scheme 2.

### 3.6. Analytical Performance of ZTO/TO MPs/GCE towards SCZ

**Figure 7:**
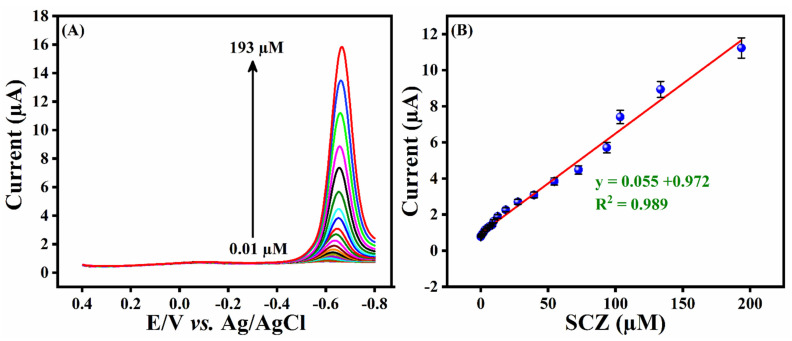
(**A**) DPVs of various concentrations of SCZ (0.01 to 193 µM) at ZTO/TO MPs/GCE (electrolyte: 0.05 M PBS, pH 7.0), and (**B**) linear plot with peak current plotted as a function of the concentration of SCZ.

The analytical performance of the ZTO/TO MPs/GCE was studied using DPV, as it is well known that this pulsed technique gives rise to lower background currents, minimising capacitive currents. The DPV data were recorded in the potential window from +0.4 to –0.8 V, and were used to determine the lowest concentration of SCZ that could be detected. The DPV curves recorded for ZTO/TO MPs/GCE after the stepwise addition of different concentrations of SCZ from 0.01 to 193 µM are displayed in Figure 7A. Upon increasing the concentration of SCZ, there is a clear increase in the peak reduction current. After plotting the peak reduction current as a function of the SCZ concentration, a linear relationship is observed over the concentration range from 0.01 to 193 µM. The corresponding linear plot is shown in Figure 7B, with a linear regression equation of *y* = 0.055*x* + 0.972 and a correlation coefficient of R^2^ = 0.989. Using this linear calibration plot, the sensitivity was computed as 0.055 µA/µM. The limit of detection (LOD) was calculated using Equation (3), where *σ* corresponds to the standard deviation of the blank signal, and S is the value of the slope obtained from the linear regression.

LOD = (3*σ*/S) (3)

Employing Equation (3), the LOD of the ZTO/TO MPs/GCE in the detection of SCZ was estimated as 0.0054 µM. These analytical parameters, including linear region (which extends from 0.01 to 193 µM), LOD, and sensitivity, compare very favourably and comparatively better than the previously reported analytical data measured using cathodic adsorptive stripping voltammetry [23] and polarography [17], as illustrated in Table 1.

**Figure 8:**
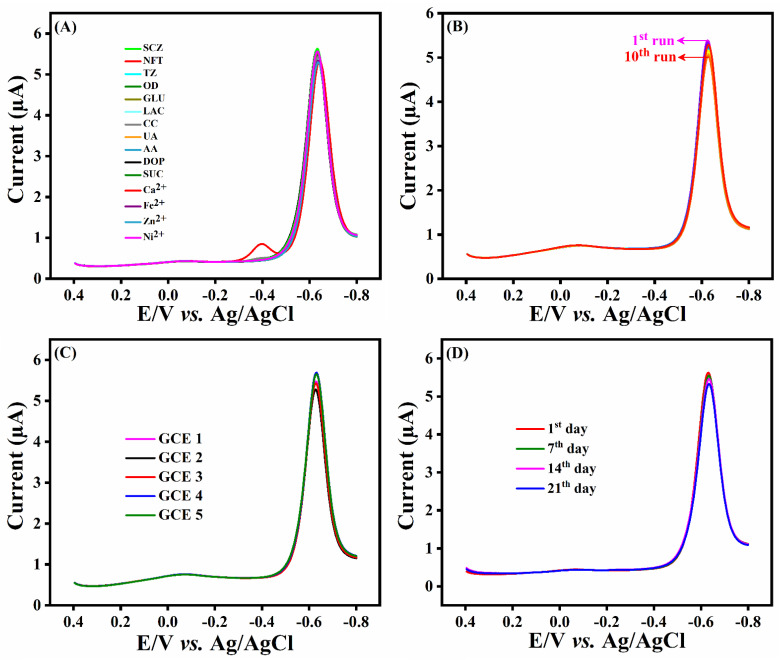
(**A**) Selectivity studies at ZTO/TO MPs/GCE for 50 μM SCZ. (**B**) Repeatability, (**C**) reproducibility, and (**D**) storage stability studies for 50 μM under an N_2_ atmosphere in pH 7.0.

The analytical performance of the ZTO/TO MPs/GCE was studied further by considering the anti-interference and selectivity, reproducibility, repeatability, and stability. To study the selectivity of the newly constructed ZTO/TO MPs/GCE sensor, the DPV technique was applied in 0.05 M PBS (pH 7.0) containing 50 µM SCZ, in the absence and presence of a number of coexisting and co-interfering biological compounds. These compounds included dopamine (DOP), lactose (LAC), catechol (CC), uric acid (UA), glucose (GLU), and sucrose (SUC)), metal ions (Ca^2+^, Fe^2+^, Zn^2+^, and Ni^2+^), and nitro group-containing drugs (nitrofurantoin (NFT), ornidazole (OD), and tinidazole (TZ)). The concentration of these potential interferents was maintained at 50 µM, giving equimolar concentrations of SCZ and the interferent. The influence of these added interferents is summarized in Figure 8A, where it is clearly observed that ZTO/TO MPs/GCE exhibits a well-defined peak for the reduction of SCZ in the presence of all of the interferents. Interestingly, the reduction of nitrofurantoin is seen in Figure 8A at about –0.4 V; however, this wave is sufficiently well separated from the SCZ reduction wave and does not contribute to the current measured for the reduction of SCZ. All of the interfering compounds have negligible effects on the peak current recorded for the reduction of SCZ with the relative standard deviation (RSD) of ≤ 2.01%. This indicates very good selectivity, which may be connected with the adsorption of SCZ at the ZTO/TO MPs/GCE partially blocking the interferents, especially the structurally related nitro-based compounds.

In Figure 8B, the data recorded in a repeatability study performed with 50 µM SCZ in 0.05 M PBS using 10 consecutive DPV measurements on a single ZTO/TO MPs/GCE are presented. The results show that ZTO/TO MPs/GCE exhibits a very good repeatability with a RSD value of 2.22%. To investigate the reproducibility of ZTO/TO MPs/GCE, five different ZTO/TO MPs-modified GCEs were prepared and employed in the detection of SCZ with DPV. Again, the obtained DPV curves, shown in Figure 8C, are very similar, with almost the same peak currents. The RSD was computed at 3.04%, demonstrating a very good reproducibility. The stability of the ZTO/TO MPs/GCE was studied following storage over a 21 day period. The constructed ZTO/TO MPs/GCE was stored and subjected to DPV analysis at seven-day intervals. In Figure 8D, the DPV curves recorded in 50 µM SCZ following 1, 7, 14, and 21 days of storage are compared. After 21 days, the ZTO/TO MPs/GCE retained 97% of its original current, indicating very good stability over a 21-day storage period.

It is clearly evident from this analysis that the developed ZTO/TO MPs/GCE exhibits a desirable selectivity, impressive reproducibility, storage stability, and repeatability, making it a promising electrode for the determination of SCZ. Indeed, when comparing the four plots in Figure 8, it is evident that the variations in the selectivity, repeatability, and reproducibility are well within the RSD value of 3.04% seen for the reproducibility analysis (Figure 8C), indicating a very good performance, well within experimental errors. 

### 3.7. Practical Analysis

**Figure 9:**
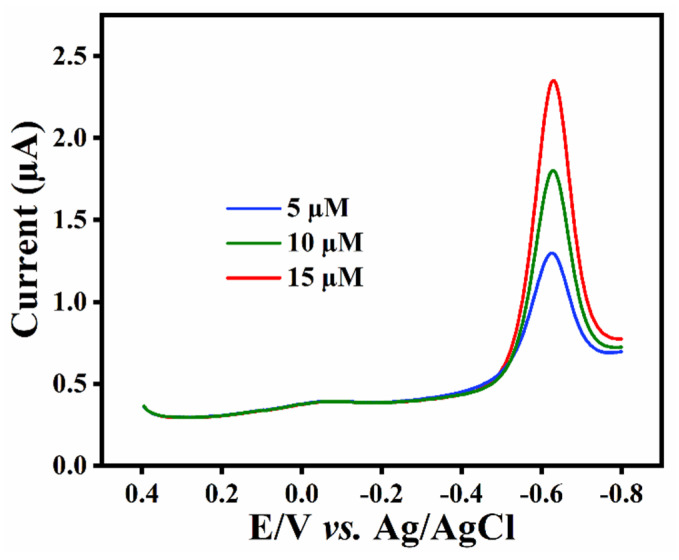
DPV curves recorded for ZTO/TO MPs/GCE in blood serum with added spikes of SCZ.

For practical applicability, a blood serum sample was collected from a medical hospital in Taiwan. Before analysis, about 5 mL of the blood serum sample was mixed with an anticoagulant (EDTA) and centrifuged at 6000 rpm for 10 min. The supernatant was collected and diluted with 10X PBS (0.05 M, pH 7.0) and designated as the stock solution. To mimic the SCZ in the stock solution, a known concentration was added to the stock solution (1:1 stock solution/SCZ ratio). Figure 9 shows the DPV curves recorded in the blood serum sample, to which a known concentration of SCZ was added using the standard addition method. The SCZ reduction waves recorded in the complex blood serum (Figure 9) were very similar to those recorded in the buffer sample (Figure 8), with the reduction peak potential and the width of the peaks being almost identical. It can be seen that the reduction peak current of SCZ at the ZTO/TO MPs/GCE increases with increasing the SCZ concentration in the blood serum sample. Good recovery results were obtained, as shown in Table 2. 

The maximum plasma concentration of SCZ in healthy individuals has been reported to reach 16.2 μg/mL [36] to 24.8 μg/mL [37], with half-life values of 19.60 and 13.86 h, respectively, following the administration of 2 g of SCZ. This is equivalent to maximum plasma levels of 87 to 133 μM. The data presented in Table 2 show that ZTO/TO MPs/GCE is a promising sensor for the analysis of SCZ in real samples, facilitating the analysis of its concentration as it decays from these maximum levels. 

## 4. Conclusions

In summary, the electrocatalytic activity of ZTO/TO MPs/GCE for the detection of the antibacterial agent SCZ was investigated using CV and DPV techniques. The ZTO/TO MPs/GCE shows higher currents than the unmodified GCE during the reduction of SCZ, and this was attributed to the adsorption of the SCZ molecules at the ZTO/TO MPs. Using the DPV technique, a linear calibration range from 0.01 to 193 µM, a LOD of 0.0054 µM, and a sensitivity value of 0.055 µA/µM were obtained for the detection of SCZ. Moreover, a very good selectivity was observed at the GCE modified with the ZTO/TO MPs using equimolar concentrations of SCZ and a number of interferents, including biological compounds, metal ions, and nitro-containing compounds. Likewise, acceptable reproducibility, repeatability, and stability results were obtained, while the modified electrode performed well in the analysis of SCZ in the blood serum. From the characterization and electrochemical results, we conclude that the ZTO/TO MP-modified GCE can be used as an efficient sensing material for the determination of the antibacterial drug SCZ.

## Figures and Tables

**Table 1:** Comparison of the analytical performance of ZTO/TO MPs/GCE in the electrochemical detection of SCZ with other sensors.

Material/Technique	Linear Range/µM	LOD/nM	Ref.
GCE/stripping voltammetry	4.0–120	1200	[23]
Hg/Polarography/CV	10–400	1000	[17]
ZTO/TO MPs/GCE/DPV	0.01–193	5.4	This work

**Table 2:** Practical analysis of SCZ in the blood serum sample at ZTO/TO MPs/GCE.

Added (µM)	Found (µM)	Recoveries (%)
5.0	5.1	103
10.0 15.0	9.7 14.6	97 97

## Data Availability

Not applicable.
